# Special Issue “Applications of Photoacoustic Spectroscopy”

**DOI:** 10.3390/molecules25215116

**Published:** 2020-11-04

**Authors:** Cristina Popa, Ana Maria Bratu, Mioara Petrus

**Affiliations:** Laser Department, National Institute for Laser, Plasma and Radiation Physic, 409 Atomistilor St., P.O. Box MG-36, 077125 Magurele, Romania

Photoacoustic spectroscopy is one of the most exciting areas of research in physics and chemistry, covering a broad range of applications from agricultural to biological, including atmospheric monitoring, space science, and air-quality measurements to security and workplace surveillance, in addition to its great potential in preclinical and clinical biomedical applications.

In this Special Issue, some representative examples of research articles focused on new developments in optical technologies for the study of photoacoustic spectroscopy applications are presented. Seven research articles and two reviews cover the topics of the Special Issue.

In recent years, photoacoustic spectroscopy has been explored for its practicality in qualitative and quantitative measurements of plant growth and development. The combination of infrared lasers with spectroscopy enabled analysis of multiple compounds simultaneously with detection sensitivity of ppb (parts-per-billion) to ppt (parts-per-trillion).

Popa et al. [1], in their article, provide a comprehensive study on the changes produced by Pb in plant respiration by monitoring gas molecules (ethylene and carbon dioxide) from plantlets’ evolution using CO_2_ laser-based photoacoustic spectroscopy for seeds germinated with Pb and compared them with the ones recorded for control plantlets. The importance of laser photoacoustic spectroscopy was used as an accurate technique, showing that lead treatment affects ethylene and carbon dioxide vapors in the respiration of plantlets by largely decreasing their concentration.

Because plant extracts are highly valuable pharmaceutical complexes recognized for their biological properties, in the same category, Negut et al. [2] studied nanostructured thin coatings containing *Anthriscus sylvestris* extract with dual bioactivity. A Fourier transform infrared spectroscopy comparative analysis was used for determining the chemical structure and functional integrity, while physical and chemical properties of laser-synthesized coatings were investigated by scanning electron microscopy. Such coatings are useful, natural, and multifunctional solutions for the development of tailored medical devices and surfaces.

Using the same Fourier transform infrared spectroscopy analysis, Dinache et al. [3] present a paper with spectroscopic studies of emulsions generated with a laser-assisted device. These results prove that the employed spectroscopy techniques are powerful tools in emulsion analysis and all these techniques show promising results in analysis of emulsions at the molecular level, such as droplet size characterization and time stability.

Asa continuation of the Dinache et al. [3] paper, in this Special Issue, Boni et al. [4] evaluated the emission of mm-sized pendant droplets containing Rhodamine 6G dye solution and dye emulsions, analyzing the geometry of excitation and collection of emitted radiation, and the effect of emulsion particles on the droplet emission. For measurements on pendant droplets, they use an experimental set-up for laser-induced fluorescence. According to the results obtained by Boni et al., these results can be used to obtain laser sources that emit in a more controlled way with different applications.

In relation with the exploration of spectroscopy, Mogaldea et al. [5] give us a study of the use of this technique in space applications. They tried to develop new oxygen-recycling technologies for astronauts’ breathing apparatus by dissociation of CO_2_ using plasma systems. The CO_2_ dissociation is highlighted through plasma characterization and carbon monoxide (CO) quantity determination. The plasma characterization was made by using an optical emission spectroscopy method (OES), and for CO, the quantity was determinate by a gas analyzer instrument. This method can be applied in space to dissociate CO_2_ and refresh the atmosphere of closed spaces.

Sensors based on photoacoustic spectroscopy (PAS) have been successfully applied in the field of environmental trace gas monitoring by Yufeng Pan et al. [6] that developed a highly sensitive nitrogen dioxide (NO_2_) photoacoustic sensor for environmental monitoring using a low-cost high-power laser diode. Yufeng Pan et al. [6] tested the outdoor sensor to detect NO_2_ concentrations in the environment. The performance of the NO_2_ photoacoustic sensor was validated by the data released by the CNEMC (China National Environmental Monitoring Center) monitoring station. The study explored the possibility to employ the NO_2_ sensor in smart traffic lights to regulate traffic flow through cities and to reduce pollution hotspots. Earlier, Pushkarsky et al. [7] reported a PAS-based sensor for NO_2_ detection at 6.25 µm by using a quantum cascade laser operating in an external grating cavity configuration, but the use of such a sensor proved to be costly and complex.

Alain Loh and Marcus Wolff [8] published the first optical detection scheme (a sensor system based on photoacoustic spectroscopy) for short-chained hydrocarbon isotopologues using multivariate analysis of photoacoustic spectra. Depending on the investigated hydrocarbon isotopologue, detection limits ranging from 0.043 ppm (parts-per-million) to 3.4 ppm, and the implementation of multivariate analysis has demonstrated that the photoacoustic spectroscopy setup works reliably and that the selective concentration determination of short-chained hydrocarbon isotopologues is possible.

Breath analysis research focused on the identification of specific biomarkers for the management of patients, particularly with regard to early diagnosis since it is non-invasive and fast [9]. Several methods for detecting breath biomarkers are currently available, such as mass spectrometry, sensor arrays, and laser-based spectroscopy [10].

In short reviews, Dumitras et al. [11] and Selvaraj et al. [12] show the potential advantages of breath analysis through the use of photoacoustic spectroscopy.

Dumitras et al. [11] reported, in their review, the applicability of laser photoacoustic spectroscopy (LPAS) in human respiration analysis. The review describes, in the first part, the fundamentals of LPAS for trace gas detection: the theoretical background, light sources, photoacoustic cell, noises, and limiting factors, and in the second part, focuses on presenting the quantification of different breath biomarkers reported by different groups using LPAS systems.

Selvaraj et al. [12] presented the recent developments in mid-infrared (MIR)laser spectroscopy which may lead to the promise of compact point-of-care. The review showed a schematic of the setup for MIR laser absorption spectroscopy and the recent advances that can lead to the development of highly compact and sensitive instrumentation for trace gas analysis. They also made a presentation of the respiratory biomarkers, such as ammonia, ethane, ethylene, acetone, nitric oxide, and methane, and the latest measurements made with the PAS technique. According to Selvaraj et al., MIR spectroscopy requires improvements in order to be implemented in the medical clinic as a tool for detecting gaseous biomarkers in the exhaled breath, but in the near future, MIR detection systems can be used to distinguish between healthy and diseased individuals.

In conclusion, this Special Issue, “Applications of Photoacoustic Spectroscopy”, reported a collection of high-quality research papers which concentrate the progress made in this field. These papers contributed, with their topics and their quality, to the success of this Special Issue.

Finally, we want to thank the authors for their contributions to this Special Issue, all the reviewers for their work evaluating the submitted manuscripts, and the editorial staff of Molecules, especially to the Assistant Editor of the Journal, Ms. Tina Li, for her kind assistance during the preparation of this Special Issue.
